# Real-world effectiveness of fidaxomicin in patients at high risk of *Clostridioides difficile* recurrence

**DOI:** 10.1017/ash.2024.381

**Published:** 2024-09-11

**Authors:** Benjamin Colwell, Jennifer Aguilar, Frances Hughes, Pavel Goriacko, Victor Chen, Mei Chang, Rachel Bartash, Yi Guo

**Affiliations:** 1 Department of Pharmacy, Montefiore Medical Center, Bronx, NY, USA; 2 Department of Pharmacy, UC San Diego Health, San Diego, CA, USA; 3 Division of Infectious Diseases, Department of Medicine, Montefiore Medical Center, Bronx, NY, USA

## Abstract

**Objective:**

Compare the real-world impact of fidaxomicin (FDX) and vancomycin (VAN) on *Clostridioides difficile* infection (CDI) recurrence in a high-risk patient population.

**Design:**

A retrospective, matched-cohort study evaluating hospitalized patients with CDI from January 1, 2016, to November 1, 2022, within a tertiary academic medical center.

**Patients:**

Adult patients with at least 1 prior CDI case who received either FDX or VAN for non-fulminant CDI while admitted, and had at least 1 additional risk factor for recurrence. Risk factors included age >70, solid organ or bone marrow transplant recipients, broad-spectrum antibiotic use within 30 days, or receipt of chemotherapy/immune-modulating agents within 30 days of admission. FDX and VAN patients were matched according to risk factors.

**Results:**

A total of 415 patient admissions were identified. After the exclusion of 92 patients for fulminant CDI, diarrhea from another cause, or use of VAN taper therapy, and 15 unmatched patients, 308 patient admissions were included (68 FDX and 240 VAN patients). There were no significant differences in 4-week recurrence (26% vs 23%; OR 1.1; *P* = .51), 90-day CDI readmission (29% vs 23%; *P* = .65), or 90-day all-cause readmission (54% vs 53%; *P* = .91). There was a significant 17% decrease in 90-day mortality associated with the use of FDX (OR .3; *P* = .04).

**Conclusions:**

In a real-world high-risk patient population, the use of FDX compared to oral VAN did not result in decreased CDI recurrence within 4 weeks or fewer hospital readmissions within 90 days. Further research is needed to better assess the value of FDX in this patient population.

## Introduction

*Clostridioides difficile* infection (CDI) is classified as an urgent threat according to the 2019 Centers for Disease Control and Prevention (CDC) antibiotic resistance threats report.^1^ CDI is the most common healthcare-acquired infection, is associated with significant healthcare costs, and has high rates of recurrence. It is estimated that 1 of 6 patients will have a recurrent infection within 2–8 weeks and 1 in 11 patients over the age of 65 with healthcare-acquired CDI will die within 1 month.^2^ As such, both the CDC and Centers for Medicare & Medicaid Services encourage antibiotic stewardship programs and have placed financial incentives for hospitals to reduce their rate of CDI.^1^

Over the past decade, there has been a shift in CDI treatment recommendations. Although all major CDI guidelines suggest either fidaxomicin (FDX) or oral vancomycin (VAN) as first-line agents, the approach for recurrent infections is largely based on expert opinion.^3–6^ Randomized, controlled trial data support the use of FDX based on similar CDI cure rates and improved sustained response after 4 weeks posttreatment.^7–11^ These data, however, predominantly reflect initial infection in patient populations with guaranteed outpatient access to FDX and few high-risk factors for recurrence. In a real-world setting, inadequate outpatient accessibility to FDX due to high cost and poor insurance coverage may lead to treatment failure. There is also scarce data specific to high-risk patient populations, including patients with cancer, transplant recipients, the elderly, and those with severe infection.

Studies published to date yielded mixed results. The only randomized analysis of immunocompromised patients with CDI was an *a posteriori* subgroup analysis of 183 cancer patients from 2 FDX approval trials.^12^ The study found a trend toward increased clinical cure and significantly improved sustained response with a 16% lower 4-week recurrence rate compared to VAN. A 2014 case series found that 90% of cancer patients with 2 or more CDI recurrences showed a sustained response with FDX.^13^ However, a retrospective review of 59 transplant recipients saw no difference between FDX and VAN in 8-week recurrence.^14^ Based on data suggesting possibly improved outcomes with FDX, we hypothesized that using FDX as the first-line agent for CDI in patients at high risk of recurrence would lead to a 15% reduction in 30-day recurrence as compared to VAN.

## Methods

### Study design and matching

This retrospective, multicenter, matched-cohort observational study identified patients with confirmed CDI who were treated with a minimum of 3 days of FDX or oral VAN within a large tertiary academic medical center comprising 4 distinct campuses in the Bronx, New York. Adult patients were eligible for matching if they had at least 1 prior laboratory-confirmed CDI case and had at least one of the following risk factors for recurrence: age of at least 70 years old, history of solid organ or hematopoietic stem cell transplant, severe infection, broad-spectrum antibiotic use within 30 days of admission, or receipt of chemotherapy or immune-modulating agents within 30 days of admission. CDI cases were confirmed using a reflex approach first utilizing glutamate dehydrogenase and toxin assays, followed by confirmatory polymerase chain reaction testing if results were discordant.

Patients were excluded if they had fulminant CDI (ileus, toxic megacolon, or hypotension/shock per IDSA^6^), received a pulsed/tapered CDI regimen, or received combination therapy to treat CDI. Metronidazole was allowed to be given if prescribed for another indication. Severe CDI was defined as the presence of leukocytosis, hypoalbuminemia, or acute kidney injury. CDI treatment was initiated during the patient’s admission and was allowed to be completed outpatient. Patient adherence data for outpatient treatment was unavailable. Fidaxomicin recipients were matched with VAN recipients on the number of risk factors, number of previous CDI cases, and year of admission using coarsened exact matching. This study was approved by the Institutional Review Board.

### Outcomes

The primary outcome was CDI recurrence within 4 weeks after treatment completion in an intent-to-treat population. Recurrent infection was defined as clinical diarrhea with either laboratory-confirmed CDI or an additional course of treatment for presumed infection leading to symptom improvement. Secondary outcomes included 90-day CDI readmission, 90-day all-cause readmission, and 90-day all-cause mortality. Readmissions were deemed related to CDI based on chart review and problem list based on ICD-10 terminology.

### Statistical analysis

To achieve 90% power, we calculated a total target enrollment of 327 patients (82 FDX patients and 245 VAN patients) to detect a 15% difference in recurrence rate. The expected difference was based on previous randomized controlled trials and an estimated global cure rate of 60% in our high-risk patient population treated with VAN. Outcome data utilized χ^2^ analysis with a significance level of .05. Baseline characteristics were analyzed using the McNemar test for categorical data and Wilcoxon rank sum test or Kruskal–Wallis for continuous data. Sensitivity analyses were conducted by adjusting outcome data for baseline confounder imbalances using logistic regression.

## Results

**Figure 1.**
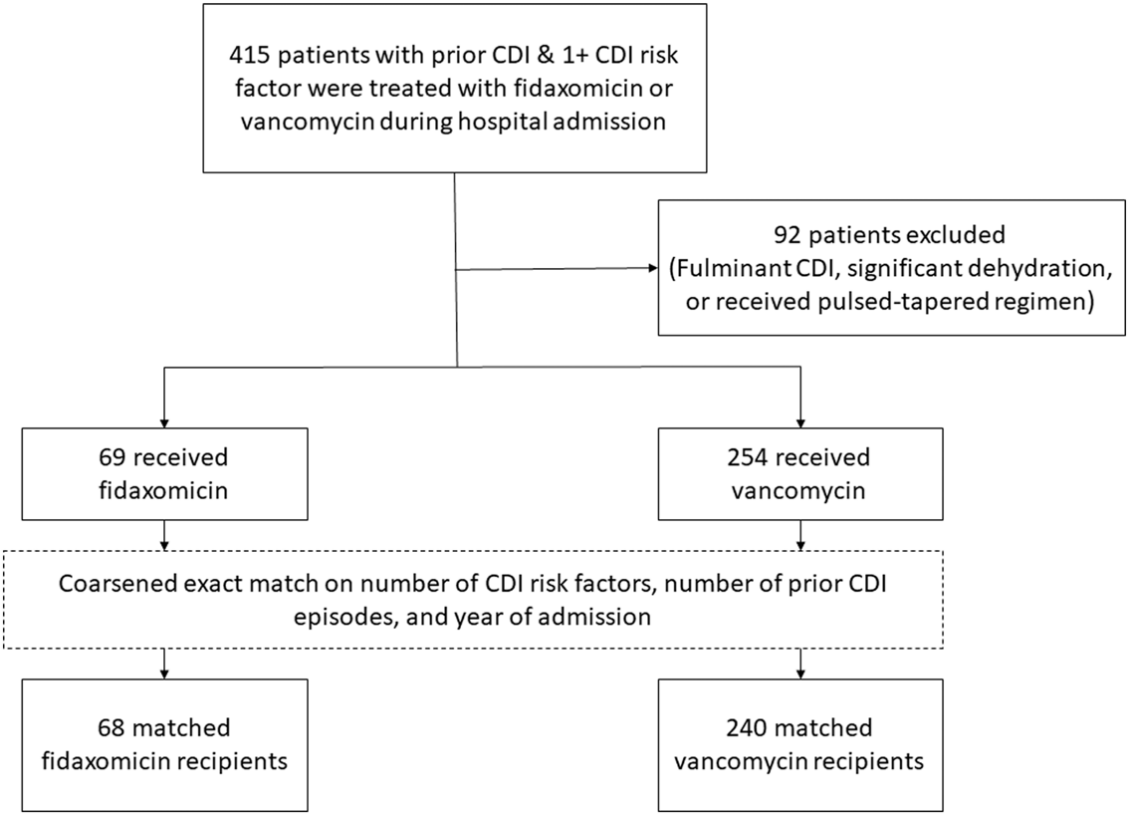
Intent-to-treat patient inclusion consort diagram.

Between January 1, 2016, and November 1, 2022, 415 hospital encounters were evaluated for inclusion. Ninety-two admissions were excluded from matching due to the use of tapered regimens or fulminant CDI. One FDX admission was excluded due to a lack of matching VAN encounters. The treatment arm contained 68 patients who received FDX 200 mg twice daily during admission. The comparator arm included 240 patients who received VAN 125 mg 4 times daily by mouth (see Figure 1).

Baseline patient characteristics are shown in Table 1. Patients were well matched with respect to age, risk factors for CDI recurrence, number of risk factors present, and number of prior recurrences. Most patients were female. The median age in both arms was 69 years old, with almost half meeting high-risk age criteria (48% FDX and 44% VAN). Over 90% of patients in both groups received antibiotics within 30 days of admission. The majority of patients met the criteria for severe CDI (60% FDX vs 65% VAN). A quarter of patients had more than 2 risk factors present upon admission.

**Table 1.** Baseline patient demographics

Characteristic	Fidaxomicin (N=68)	Vancomycin (N=240)	*P*
Age, median years (IQR)	69 (58–78)	69 (58–77)	0.856
Male sex, n (%)	17 (25)	89 (38.2)	0.07
Hospital LOS, median days (IQR)	9 (5–16)	8 (5–15)	0.536
Treatment duration, median days (IQR)	10 (7–11)	10 (7–14)	0.357
>1 CDI recurrence, n (%)	16 (23.5)	56 (23.5)	1
Risk factors, n (%)			
Transplant recipient (SOT or BMT)	12 (17.6)	32 (13.4)	0.487
Age > 70 yr	33 (48.5)	105 (44.1)	0.578
Antibiotic use within the prior 30 d	64 (94.1)	220 (91.8)	0.546
Antineoplastic/immune-modulating agent within the prior 30 d	10 (14.7)	36 (15.4)	0.913
Severe CDI	41 (60.3)	156 (65.1)	0.542
>2 risk factors, n (%)	18 (26.5)	67 (27.9)	0.845
Concurrent infection, n (%)	17 (25.4)	113 (49.7)	0.002
Concurrent antibiotics, n (%)	19 (28.4)	123 (53.6)	0.001
Received other CDI antibiotics during admission, n (%)	8 (11.9)	41 (18.0)	0.29
Hemodialysis, n (%)	9 (13.4)	49 (21.1)	0.208
Irritable bowel disease, n (%)	6 (9.0)	4 (2.0)	0.009

Note. CDI, *Clostridioides difficile* infection; IQR, inter-quartile range; LOS, length of stay; SOT, solid organ transplant; BMT, bone marrow transplant.

Results of primary and secondary outcomes are shown in Table 2. No difference was observed in 4-week recurrence rates between those receiving FDX and VAN (26% vs 23%; OR 1.1; *P* = .51). There was also no significant difference in the rate of CDI readmission at 90 days after treatment completion or 90-day all-cause readmission. We did observe that the FDX arm had a 17% lower 90-day all-cause mortality, which was statistically significant (OR 0.3; *P* = .04).

**Table 2.** Primary and secondary outcome data

Clinical end point	Fidaxomicin, n (%)	Vancomycin, n (%)	*P*
*Primary*			
CDI recurrence at 4 wk	17 (26)	53 (23)	0.51
*Secondary*			
90-d CDI readmission	19 (28)	53 (23)	0.65
90-d all-cause readmission	36 (54)	125 (53)	0.91
90-d all-cause mortality	8 (12)	67 (29)	0.04

Note. CDI, *Clostridioides difficile* infection.

When adjusting for imbalances in baseline patient characteristics, which included the patient’s sex, concurrent antibiotics, concurrent non-CDI infection, and presence of irritable bowel disease, the findings were consistent with the primary analyses. There were no differences in 4-week recurrence rates between FDX and VAN treatment (OR 1.1; *P* = .083), while the 90-day mortality was significantly lower in the FDX group (OR 0.3; *P* < .01).

## Discussion

Guidelines either recommend FDX as first-line treatment for CDI or consider FDX and VAN to be interchangeable.^3–6^ Considering the financial barriers of FDX, this study sought to determine the potential benefits of FDX as a first-line option in patients at high risk for recurrent CDI, by evaluating the efficacy of FDX in a real-world setting at an institution serving an underprivileged population with many patients at high risk for recurrent infection.

Our study did not find a difference in the primary outcome, as both FDX and VAN had similar 4-week posttreatment recurrence rates of ∼25%. The recurrence rate of VAN is consistent with Cornely et al’s findings that 26.4% of patients randomized to receive FDX did not experience a 30-day sustained response.^12^ Our 90-day CDI readmission rate of 28.4% is slightly lower compared to Guery et al’s findings that 33.1% of patients with 2 or more recurrent CDI failed to have a 90-day sustained response.^10^ Our lack of difference in CDI recurrence is consistent with findings by Clutter et al, although they evaluated an exclusively transplanted population.^14^ Recent preliminary retrospective data of 238 immunocompromised patients treated for CDI with either FDX (n = 38) or VAN (n = 200) from 2011 to 2021 also identified no difference in the secondary outcomes of 30-day CDI relapse or CDI relapse on days 31–90.^15^ We also detected no difference in readmission rates.

Although the 90-day mortality outcome was statistically significant in both primary and sensitivity analyses, its clinical impact is difficult to interpret. The study was not powered to detect differences in mortality. There are also possible confounding factors that may have contributed to mortality unrelated to CDI. Given the baseline discrepancy in concurrent infection and use of broad-spectrum antibiotics, it is possible that patients prescribed VAN were of higher acuity. Although 90-day mortality is more frequently described in prior literature, either CDI-confirmed mortality or 30-day mortality may have been more appropriate metrics for this retrospective review.

There are multiple strengths to this study. To our knowledge, this contains the largest evaluation of FDX use in high-risk patients to date. Prescribing CDI antibiotics requires infectious disease service approval at our institution, which helps minimize variance in prescribing practices. The median prescribed treatment duration in both FDX and VAN arms was 10 days, which reflects the standard of care. Additionally, matching by year of admission limits temporal bias in prescribing practices. Finally, we employed rigorous confounder adjustment methods, which included exact matching on pre-specified confounders, as well as a post hoc adjustment for any additional baseline imbalances.

This study has a few key limitations. The final outcome analysis is slightly underpowered, which makes it difficult to interpret the lack of statistical difference between recurrence and readmissions. The study did not track the incidence of NAP1 strain CDI. Data collector inter-rater variability may have affected the interpretation of the exclusion criteria. No patients received bezlotoxumab, so this study cannot evaluate concomitant use of bezlotoxumab in patients with refractory CDI. Finally, the retrospective design introduces the potential for missing documentation. This prevented accurate analysis of global cure and makes it impossible to draw firm conclusions on whether limited FDX outpatient availability could have contributed to the higher recurrence rates seen in this study.

In a real-world high-risk patient population, the use of FDX compared to oral VAN did not result in decreased CDI recurrence within 4 weeks or fewer hospital readmissions within 90 days. As of this publication, this study contains the largest retrospective review of patients with multiple risk factors and multiple recurrent CDI. More information is needed in this patient population to help justify the efficacy and cost of FDX over oral VAN.

## Research transparency and reproducibility

In the interests of supporting transparency and openness, all data, methodologies, and analysis utilized in this study will be made available upon request as appropriate to facilitate scrutiny and replication by fellow researchers and interested parties.
